# Draft genome sequence of *Psychrobacter nivimaris* LAMA 639 and its biotechnological potential

**DOI:** 10.1016/j.dib.2022.107927

**Published:** 2022-02-05

**Authors:** Brendon Egon Kormann Staloch, Henrique Niero, Robert Cardoso de Freitas, Patricia Ballone, Fernanda Rodrigues-Costa, Daniela Barretto Barbosa Trivella, Andréa Dessen, Marcus Adonai Castro da Silva, André Oliveira de Souza Lima

**Affiliations:** a School of Sea, Science and Technology, University of Vale do Itajaí (UNIVALI), 88302-202, Itajaí-SC, Brazil; b Brazilian Biosciences National Laboratory (LNBio), National Center for Research in Energy and Materials (CNPEM), 13083-100, Campinas-SP, Brazil; c Faculty of Pharmaceutical Sciences, University of Campinas (UNICAMP), 13083-871 Campinas-SP, Brazil; d Institute of Biology, University of Campinas (UNICAMP), 13083-862 Campinas-SP, Brazil; e Univ. Grenoble Alpes, CEA, CNRS, Institut de Biologie Structurale (IBS) F-38044, Grenoble, France

**Keywords:** Psychrobacter, Extremophile, Whole-genome sequencing, Illumina, Enzyme exploiting

## Abstract

Bacteria of the genus *Psychrobacter* are known for their psychrophilic characteristics, being extremophilic organisms capable of surviving and reproducing in hostile environments of low temperature and high pressure. Among many of the genus characteristics, there is the ability to produce enzymes and molecules of industrial biotechnology importance, such as pigments and proteins related to heavy metal bioremediation. The bacterium strain *Psychrobacter nivimaris* LAMA 639 was isolated from sediments from the Walvis Ridge ocean crest at a depth of 4.400 m (33.40 S 2.35 E). It is a nonmotile, halotolerant, cream-colored gram-negative aerobic bacterium. Its cultivation was performed in marine agar plates and inoculated into test tubes with NaCl at an optimal temperature of 30 °C and with shaking at 100 rpm. Genome extraction was performed with the DNeasy Blood & Tissue Kit (QIAGEN®). Sequencing was performed by Macrogen using the NovaSeq® 6000 platform (Illumina) applying the whole genome shotgun (WGS) method. Thereafter, 14.712.526 reads of 151 bp were generated, totaling 2.2 G bp with a GC content of 42.9%. Assembly and mapping were performed with a CLC Genomics Workbench. The best assembly considered was the one with the lowest number of contigs and the highest base length pair. The assemblies were evaluated using QUAST, and the best resulting variant was selected for annotation. Genome annotation was performed with RAST and PATRIC; the antiSMASH tool was used for secondary metabolites; NaPDoS was used for domains; and three-dimensional structural prediction of relevant proteins was performed using Phyre2. Annotation with ClassicRAST generated 2,891 coding sequences (CDSs) distributed in 402 subsystems. Annotation with PATRIC generated 2,896 coding sequences, among them 776 hypothetical proteins. The antiSMASH tool visualized a beta-lactone cluster in contig 06. In the search for natural products with NaPDoS, two ketosynthase domains were identified. The search for relevant proteins was performed using the AMFEP list as a criterion. From these data, 34 possible enzymes with biotechnological potential were found. Finally, the organism is presented as a new reference regarding the potential of deep-sea marine bacteria, demonstrating that, from the annotated and cured genome, it is possible to find in its genetic repertory products of interest for biotechnological applications.

## Specifications Table

**Table:** 

Subject	Omics: Genomics
Specific subject area	Bacterial Genomics, Applied Microbiology and Biotechnology
Type of data	Draft genome sequence data, figures, tables
How data were acquired	Whole-genome sequencing on a NovaSeq 6000 platform (Illumina). The genome was assembled with CLC Genomics Workbench (v. 6.5.2) and annotated with QUAST, RAST, PATRIC, antiSMASH, NaPDoS and Phyre2.
Data format	Raw, analyzed and assembled genome sequences
Parameters for data collection	Genomic DNA was extracted from a pure culture of LAMA 639 isolated; DNA library preparation; whole genome sequencing; de novo assembly; annotation RAST and PATRIC.
Description of data collection	Genomic DNA extraction was performed from a pure culture of *Psychrobacter nivimaris* LAMA 639 using a DNeasy Blood & Tissue Kit (QIAGEN®); library was prepared using a TruSeq Nano DNA (350) for Illumina®; sequencing was performed using a NovaSeq® 6000 Illumina system. The genome was assembled using CLC Genomics Workbench (v 6.5.2), annotated using Rapid Annotation using Subsystems Technology (RAST) and Pathosystems Resource Integration Center (PATRIC).
Data source location	Universidade do Vale do Itajaí (UNIVALI), Itajaí, Santa Catarina, Brazil. *Psychrobacter nivimaris* strain LAMA 639 was isolated from sediments (depth 4.400 m) from the Atlantic Ocean - Walvis Ridge (33.40 S 2.35 E).
Data accessibility	A sequence of 16S rRNA has been deposited in the NCBI GenBank under accession number JX860208.1. Direct link to data: https://www.ncbi.nlm.nih.gov/nuccore/JX860208.1 Raw reads have been deposited in the NCBI Sequence Read Archive (SRA) under accession number: SRX13085745. Direct link to data: https://www.ncbi.nlm.nih.gov/sra/PRJNA557251 The draft genome sequence has been deposited in GenBank under accession number NZ_VZIZ00000000. The direct URL to the data is https://www.ncbi.nlm.nih.gov/nuccore/NZ_VZIZ00000000.1. BioSample ID in GenBank are SAMN12392404 (https://www.ncbi.nlm.nih.gov/biosample/SAMN12392404). Genome ID in PATRIC is 281,738.12 (https://www.patricbrc.org/view/Genome/281738.12). All additional data analysis files and supplementary tables can be accessed at Mendeley Data, V1, doi: 10.17632/dbvc4fth8f.1 (https://data.mendeley.com/datasets/dbvc4fth8f/1)

## Value of the Data

• The draft genome sequence of *P. nivimaris* LAMA 639 provides fundamental knowledge of this organism and insight for biotechnological applications in many different areas.
• Data from this draft genome can be useful for comparative genomic analyses of *Psychrobacter* species and will be useful for further functional genomics and enzyme engineering research.
• The draft genome sequence of *P. nivimaris* LAMA 639 can help elucidate the mechanism of survival of extremophile organisms in hostile environments. In addition, it can be used in genomic comparisons and enzyme production.

## 1 Data Description

**Fig. 1:**
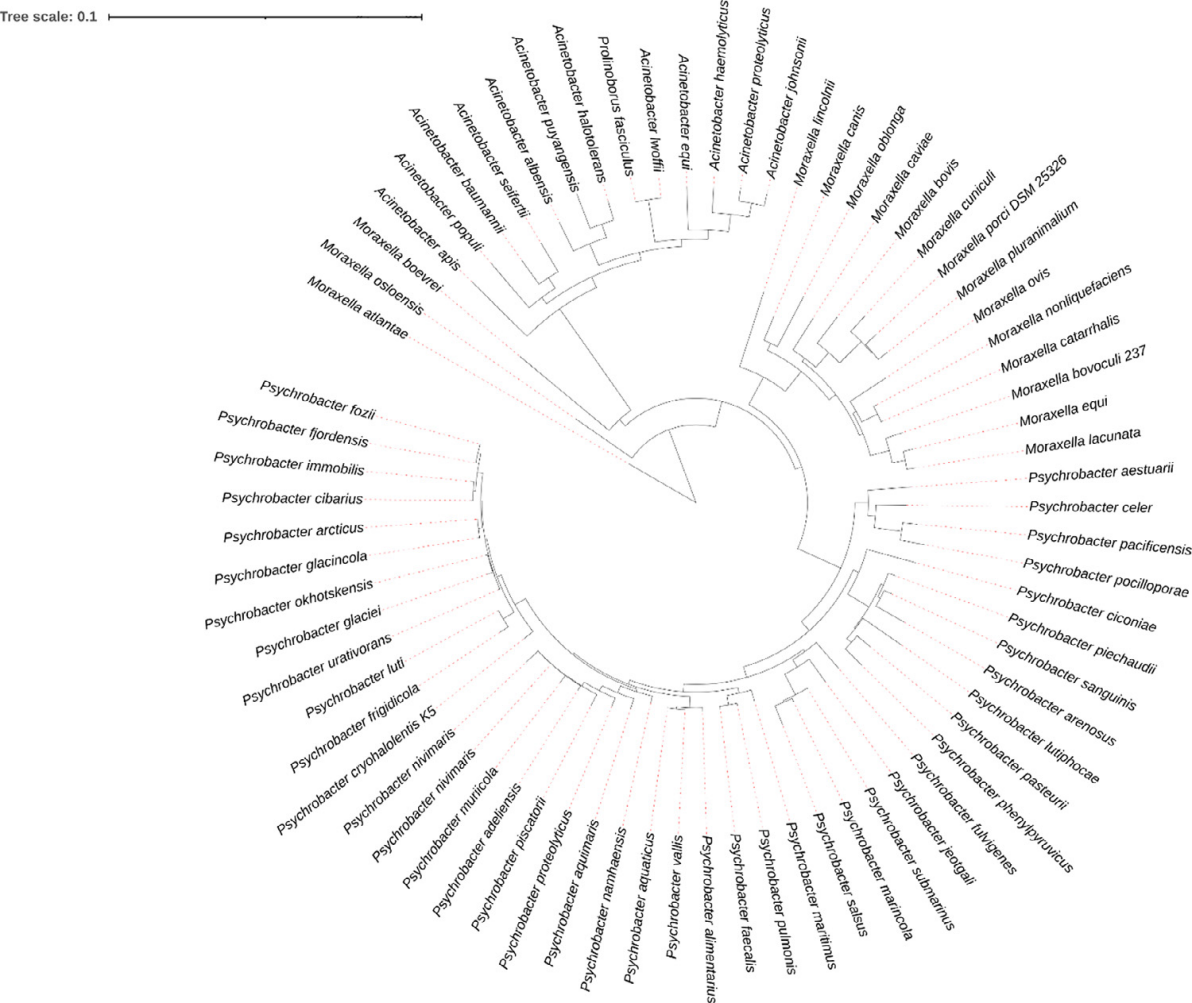
Neighbor-joining tree based on the 16S rRNA sequence showing the relationships between strain LAMA 639 and its related type strains. The 71 closest strains were used. The maximum sequence difference allowed was 0.75.

**Fig. 2:**
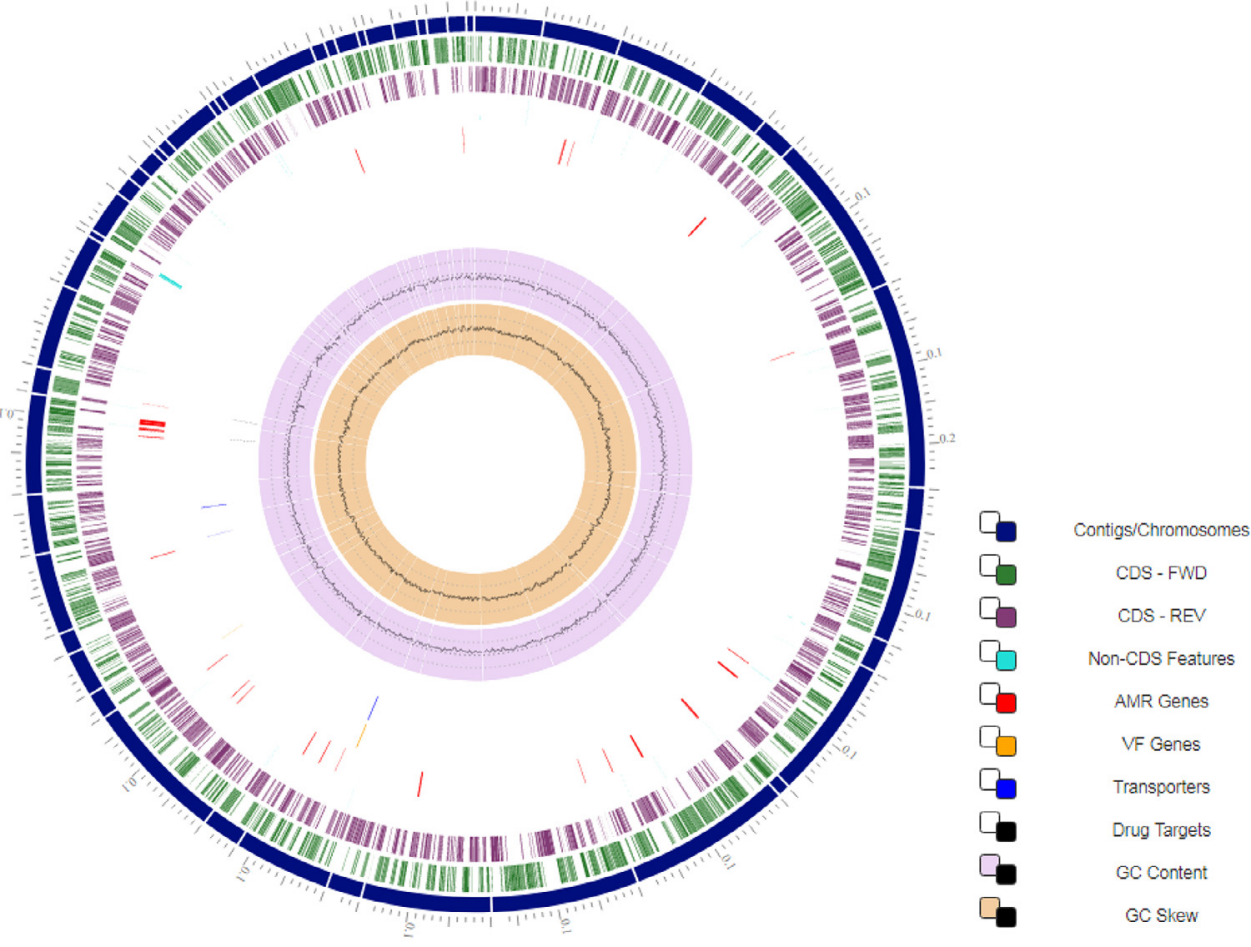
Circular map of the genome of Psychrobacter nivimaris LAMA 639. From outermost to innermost data: contigs (dark blue); forward coding sequences (green); reverse coding sequences (purple); antimicrobial genes (red); virulence factors genes (orange); transporters (blue); drug targets (black); GC content (lilac); GC skew (orange). The tool used to visualize the circular genome was PATRIC.

*Psychrobacter nivimaris* is an aerobic, gram-negative, nonmotile bacterium that grows in cream-colored colonies at temperatures ranging from 5 to 35 °C [1]. The genus *Psychrobacter* is known for its psychrotolerant and psychrophilic characteristics, that is, they are extremophile bacteria capable of living and reproducing in hostile environments of low temperatures and high pressures [2]. These characteristics are highly valued in bioprospecting relevant and biotechnologically interesting molecules, as shown by the annual review of marine natural products (MNPs) [3]. It is now worth citing that most MNPs from macroinvertebrates can be produced directly by microorganisms, reducing investment and technical effort in other taxonomic groups. The *Psychrobacter nivimaris* LAMA 639 strain described in this study was isolated from Walvis Ridge ocean crest sediments at a depth of 4.400 m (33.40 S 2.35 E) [4]. Phylogenetic analyses based on 16S rRNA gene sequences revealed that the strain is close to the *Moraxella* and *Acinetobacter* genera, exhibiting more than 99% similarity with *P. muriicola* strain 2pS and *P. adeliensis* strain DSM 15,333 (Fig. 1). Previous studies of a whale carcass microbiota demonstrated the possibility of identifying economically and industrially relevant enzymes, such as lipases, esterases, galactosidases, glucosidases and amylases [5]. Therefore, this bacterium was selected for genome sequencing and subsequent prospecting of genes with biotechnological relevance (Table 1). Genome sequencing returned 50 contigs of 3.285.420 bp in total length. The longest contig had 252.504 bp and presented an N50 of 117.043 bp. The characteristics of the *P. nivimaris* LAMA 639 genome are illustrated in Fig. 2. The G + C content of 42.9% was close to that typically found in *Psychrobacter* spp., which averages 43.26%. CheckM software was used to verify the quality and reliability of the genomic data set obtained, determining the estimated genome completeness at 99.11% and the estimated contamination at 1.93%, which characterize a high-quality genome (>95%) and low contamination (<5%), respectively [6]. The best assembly was considered with TRIM of Q25, word size of 61, overlap length of 60% and similarity fraction of 80%. The main attributes of genome assembly and annotation are summarized in Table 2.

**Table 1:** Project features and general information of Psychrobacter nivimaris strain LAMA 639 according to MIGS recommendations [7].

Property	Term	Evidence code*
Geographic location	Walvis Ridge	TAS
Latitude	33.40 S	TAS
Longitude	2.35 E	TAS
Depth	4400 m	TAS
Time of sample collection	November 2009	TAS
Habitat	deep-sea sediment	TAS
Number of replicons	1	TAS
Extrachromosomal elements	0	TAS
Reference for biomaterial	https://dx.doi.org/10.1186%2F2193–1801–2–127	TAS
Source material identifiers	Still not deposited	
Pathogenicity	Non-pathogenic	TAS
Biotic relationship	Free-living	TAS
Specific host	Environmental	TAS
Trophic level	Heterotrophic	TAS
Oxygen requirement	Aerobic	TAS
Isolation and growth conditions	Isolated in Zobell Marine Broth medium 2216 at 10 °C	TAS
Nucleic acid preparation	Extraction with DNeasy Blood & Tissue Kit (QIAGEN®)	IDA
Sequencing method	150 bp paired-end sequencing reads	IDA
Assembly	De novo assembly based on de Bruijin graphs	IDA
Finishing quality	Draft sequence	IDA
Sequencing platforms	Illumina NovaSeq 6000	IDA
Fold coverage	670x	IDA

⁎ Evidence codes - IDA: inferred from direct assay; TAS: traceable author statement.

**Table 2:** Main characteristics and statistics of LAMA 639 draft genome assembly and annotation.

Feature	Value (CLC)	Value (NCBI)*
Contig count	50	54
Scaffold	–	52
Total contigs length (bp)	3285,420	3282,829
Number of N's (uncalled bases)	272	–
N50	117,043	97,373
N75	64,631	–
L50	10	11
L75	19	–
Maximum contig length (bp)	252,504	252,506
Average contig length (bp)	67,046	63,131
G + C content	42.88	42.85
rRNA genes	3	3
tRNA genes	41	42
CDS (RAST annotation)	2887	2819
Completeness	99.11%	–
Contamination	1.93%	–

⁎ NCBI submission parameters. Any stretch of 10 or more Ns in a sequence is treated as a gap between two contigs in a scaffold when counting contigs and calculating contig N50 & L50 values.

**Fig. 3:**
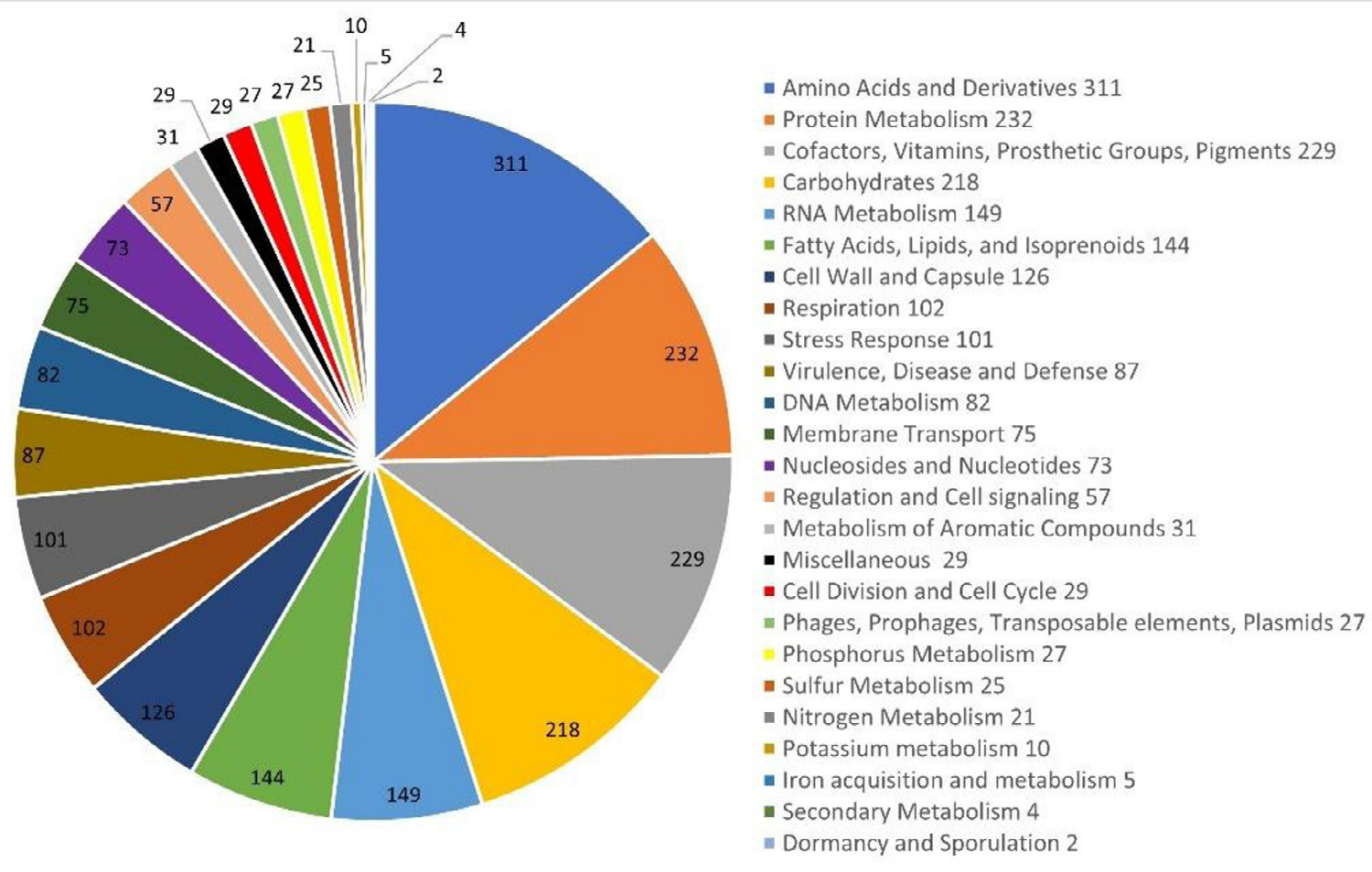
Overview of the subsystem categories assigned to the genome of Psychrobacter nivimaris LAMA 639, in descending order. The genome assembly was annotated using the RAST server.

Genome annotation was performed using the Rapid Annotation Subsystem Technology (RAST) [8], which generated 2887 gene coding sequences (CDSs) divided into 402 subsystems (Fig. 3). Most of the CDSs were assigned to subsystems related to amino acids and derivatives (311), to protein metabolism (282), to cofactors, vitamins, pigments and prosthetic groups (229) and to carbohydrates (218). Of the 2887 protein-coding genes of the LAMA 639 strain, 18 genes were associated with protein degradation, 101 genes associated with the stress response (cold-shock proteins, heat shock proteins, proteins associated with osmotic stress, among others) and many genes (87) associated with cell defense, antibiotic and toxic substance resistance, including fluoroquinolone resistance genes (4). The data annotated by RAST also indicated the presence of several metabolic pathways encoded in the genome of *P. nivimaris* LAMA 639 with significant biotechnological potential.

With the goal of comparing results to the RAST annotation, another annotation was performed in the PATRIC system, which identified 2896 CDS, 2120 associated with known proteins, 1401 of which were included in a subsystem. Among the 776 hypothetical proteins identified, none were included in a subsystem. The most representative classes were genes encoding cofactors, vitamins and prosthetic groups (205), amino acids and derivatives (174), as well as genes involved in protein synthesis (174), and respiration (107). We also identified 36 genes involved in special processes, 2 genes for virulence factors, 3 transport genes, and 29 genes related to antibiotic resistance. It is important to highlight that hypothetical proteins are the main targets in the search for new proteins or enzymes with potential for biotechnological application. In regard to *Psychrobacter nivimaris*, the characteristics of psychrotolerance and barotolerance that extend to its macromolecules could also be explored [9].

Gene prospecting is closely linked to the data processing capacity of available bioinformatics tools. Currently, the production of data grows exponentially, and it is necessary that technologies keep up with this growth and be capable of quickly and efficiently interpreting them in favor of technological, sustainable and economic development. Regarding the biological prospects of enzymes with different characteristics from those already commercialized, bacteria adapted to withstand low temperatures, high pressures, a high osmotic gradient and a lack of nutrients stand out [10]. In addition, the Natural Product Reports journal published the discovery of 1554 new compounds, found just in 2018, of which 240 are from bacteria of marine origin. [3]. Therefore, bioinformatics tools were used to identify enzymes and molecules of biotechnological interest in the genome of *P. nivimaris* LAMA 639. The presence of hydrolytic enzymes (lipases, proteases, cellulases, among others), exopeptidases (aminopeptidase, d-alanyl-d-alanine carboxypeptidase), hydrolases (alpha-amylase, l-asparaginase, catalase, alpha-glucosidase, monoacylglycerol lipase, protease, triacylglycerol lipase, peroxidase, phospholipase A and B, pullulanase), esterases (phosphodiesterase) and transferases (glucosyltransferase) was exploited. Table 3 summarizes the 35 enzymes found after manual curation, which were prospected using the Enzyme Commission (EC) number and Phyre2 homology modeling, based on the list of the Association of Manufacturers and Formulators of Enzyme Products (AMFEP), which lists the main enzymes with biotechnological applications. The different application areas were determined from the BRENDA database [12]. Finally, an identity BLASTP search was performed with the Swiss-Prot database identifying the highest homology species proteins, and all E-values were considered for enzyme curation and had a relevant value.

**Table 3:** List of enzymes found in the genome of Psychrobacter nivimaris LAMA 639 demonstrating the 35 cured enzymes with potential biotechnological relevance. A BLASTP search was performed in the Swiss-prot database, visualizing the identity and to which organism the molecule belongs, providing the accession number. Finally, the number of applications that the molecule has according to the BRENDA database is also indicated.

EC	Enzyme (Recommended Name)	Size in aa	Identity (%)	Organism	Acession	Applications
1.1.1.1	alcohol dehydrogenase	342	92.60	*Moraxella* sp.	Q8GIX7.1	62
1.1.1.1	aldehyde-alcohol dehydrogenase	434	38.52	*Escherichia coli*	P37686.4	62
1.1.1.202	1,3-propanediol dehydrogenase	387	35.46	*Geobacillus thermodenitrificans*	A4IP64.1	25
1.11.1.21	catalase-peroxidase	789	73.11	*Cellvibrio japonicus*	B3PC77.1	10
1.11.1.6	catalase	695	55.81	*SinoRhizobium meliloti*	Q9 × 576.2	31
1.15.1.1	superoxide dismutase	185	35.14	*Aquifex aeolicus*	O67149.1	30
1.15.1.1	superoxide dismutase	209	58.76	*Synechocystis* sp.	P77968.3	30
2.4.1.129	peptidoglycan glycosyltransferase	661	43.76	*Haemophilus influenzae*	P44469.1	5
2.4.1.173	sterol 3beta-glucosyltransferase	361	82.55	*Psychrobacter arcticus*	Q4FQV9.1	4
2.5.1.19	3-phosphoshikimate 1-carboxyvinyltransferase	777	61.38	*Methylococcus capsulatus*	Q608S5.2	21
3.1.1.1	carboxylesterase	287	35.48	*Pseudomonas aeruginosa*	Q9HZY8.1	71
3.1.1.101	poly(ethylene terephthalate) hydrolase	314	69.52	*Moraxella* sp.	P19833.1	45
3.1.1.23	acylglycerol lipase	318	24.43	*Mycolicibacterium smegmatis*	A0QNZ7.1	14
3.1.1.29	aminoacyl-tRNA hydrolase	193	95.34	*Psychrobacter arcticus*	Q4FVB5.1	1
3.1.1.3	triacylglycerol lipase	358	49.33	*Pseudomonas aeruginosa*	P26876.2	184
3.1.1.32	phospholipase A1	482	43.20	*Neisseria meningitidis*	Q9K0U7.1	20
3.1.1.5	lysophospholipase	318	26.11	*Homo sapiens*	Q99685.2	7
3.1.4.12	sphingomyelin phosphodiesterase	279	69.53	*Psychrobacter arcticus*	Q4FQ25.1	14
3.1.4.46	glycerophosphodiester phosphodiesterase	452	46.27	*Trichophyton benhamiae*	D4AIS9.1	8
3.4.11.1	leucyl aminopeptidase	551	47.69	*Acinetobacter baumannii*	A3M1A8.2	24
3.4.11.18	methionyl aminopeptidase	263	58.73	*E. coli*	P0AE18.1	14
3.4.11.2	membrane alanyl aminopeptidase	880	40.99	*Oryza sativa Japonica*	B7EA73.1	43
3.4.11.9	Xaa-Pro-aminopeptidase	605	35.04	*Arabidopsis thaliana*	Q8RY11.1	5
3.4.21.89	Signal peptidase I	300	38.18	*Pseudomonas aeruginosa*	Q9I5G7.1	48
3.4.23.36	Signal peptidase II	235	49.42	*Acinetobacter baylyi*	Q6FG03.1	1
3.4.23.43	prepilin peptidase	299	47.81	*Pseudomonas stutzeri*	Q9ZEL6.1	3
3.5.1.1	asparaginase	366	29.80	*Deinococcus radiodurans*	Q9RRX9.2	80
3.5.1.108	UDP-3-O-acyl-N-acetylglucosamine deacetylase	320	89.38	*Psychrobacter cryohalolentis*	Q1Q950.1	19
3.5.1.16	acetylornithine deacetylase	430	32.91	*Pasteurella multocida*	Q9CLT9.2	2
3.5.1.18	succinyl-diaminopimelate desuccinylase	399	92.07	*Psychrobacter cryohalolentis*	Q1QDC1.1	4
3.5.1.2	glutaminase	306	85.90	*Psychrobacter cryohalolentis*	Q1QB42.1	21
3.5.1.28	N-acetylmuramoyl-l-alanine amidase	291	38.57	*E. coli*	P75820.1	15
3.5.1.5	urease	729	80.39	*Psychrobacter cryohalolentis*	Q1QC36.2	34
3.5.2.6	beta-lactamase	680	20.89	*Bacillus subtilis*	P39844.1	21

Of these enzymes, 82% have applications in the area of medicine, 62% in the area of drug development and product synthesis, 47% in the area of biotechnology, 41% demonstrated applicability in diagnostic techniques, 35% can be used in pharmacology, 29% have compound degradation activity, 24% can be used both for industrial application and for food industry and, finally, 18% of them have potential for application in agriculture. Considering this, 24 hydrolases, 7 oxidoreductases and 3 transferases were located, whose sizes ranged from 185 to 880 amino acids, with an average of 435 amino acids per molecule. One notable example is asparaginase (EC 3.5.1.1), which has received special attention from scientists for its antineoplastic activity, has been studied since the beginning of the 20th century [13]. The sequence was analyzed with the BLASTP tool [14] in the Swiss-Prot database and showed a similarity of 29.8% with asparaginase from the organism *Deinococcus radiodurans* strain R1 and 51% coverage of the sequence. A BLASTP search was also performed with the patent bank (pataa), indicating 44.5% identity, an E-value close to zero and 91% coverage of the sequence with a molecule of therapeutic use. The three-dimensional homology analysis by Phyre2 [11] returned 100% confidence with an l-asparaginase in which 97% of the residues were used for modeling. These identity analyses indicate that LAMA 639 strain asparaginase has potential enzymatic activity. Another molecule that deserves attention is poly(ethylene terephthalate) hydrolase (EC 3.1.1.101), known as PET hydrolase. This enzyme is used in the treatment of PET waste, which occupies a prominent place among the current environmental problems [15]. The analysis with BLASTP found a similarity of 69.5% with the molecule of the organism *Moraxella* sp., coverage of 99% and E-value close to zero. The investigation with the patent bank indicated similarity with several enzymes that act in enzymatic degradation processes. Furthermore, the analysis with Phyre2 modeled, with 100% confidence and coverage of 80% of the residues, the three-dimensional homology between the PET hydrolase of *P. nivimaris* LAMA 639 and that of *Ideonella sakaiensis*, and indicated 43% similarity. That said, it justifies the need for further studies both with the enzymes asparaginase and PET hydrolase and with other molecules identified in the genome to verify their real applicability in different contexts. The characteristics of all curated molecules are available in the Supplementary Data, along with their sequence, three-dimensional models and number of applications in each area.

After enzyme curation, the anti-SMASH 5.0 platform was used to search for biosynthetic gene clusters (BGCs) aimed at secondary metabolites [16]. We identified a gene cluster that is located in contig 6, between 160.650 and 188.408 bp, in the genome of *P. nivimaris* LAMA 639. The gene is part of the beta-lactone group and has low similarity (15%) with the plipastatin cluster. However, this similarity does not include condensation domains necessary for the synthesis of nonribosomal peptides. Nevertheless, this low similarity may indicate a new biosynthetic pathway that has not yet been explored for this class of molecules. It is also known that this class of betalactone BGCs is present in most organisms of the *Psychrobacter* genus as the only BGC present and has known activity in producing compounds with antimicrobial activity [17].

In the search of domains of natural products, the Natural Product Domain Seeker (NaPDoS) database was used [18]. Two ketosynthase domains that are linked to polyketide biosynthesis were obtained. Prospecting biosynthetic pathways for the production of vitamins found in RAST routes for biotin, riboflavin, thiamine and pyroxidine. However, further studies are needed to assess whether the pathways are functional in the genome of the LAMA 639 strain.

## 2 Experimental Design, Materials and Methods

### 2.1 Isolation of *psychrobacter nivimaris* LAMA 639 strain

The sample used for the research of the LAMA 639 strain was isolated from Walvis Ridge oceanic crest sediments at a depth of 4400 m in an expedition to the South Atlantic Ocean [4]. The isolation temperature of the strain was 4 °C in marine agar medium.

### 2.2 Genomic DNA extraction and quantification

Aliquots (10 µL loop) of bacterial cells suspension (10^7^ cells/mL) were inoculated (streak) in Petri dishes with solid marine agar medium composed of 4% marine broth (Zobell 2216 – HiMedia) and 1.5% bacteriological agar (Vetec). Isolated colony was used for DNA purification, cultivation was carried out for 24 h at 30 °C in LB liquid medium. For DNA extraction from *P. nivimaris* LAMA 639, the commercial product DNeasy Blood & Tissue Kit (QIAGEN) was used following the manufacturer's instructions for gram-negative bacteria. Subsequently, 1% agarose gel electrophoresis was performed to verify the quantity and quality of the DNA. After electrophoresis, the sample was quantified using an Infinite 200 PRO microplate reader (TECAN) and had an average concentration of 98 ng/µL.

### 2.3 Library construction and genome sequencing

The genomic material was sent to a company specialized in sequencing (Macrogen). Using TruSeq Nano DNA (Illumina), the genomic library for whole genome sequencing was prepared, and sequencing was performed by the NovaSeq 6000 platform (Illumina). The DNA was fragmented by the shotgun method to generate paired readings of 150 bp. Thus, 14.712.526 readings were found with a total of 2,2 G bp and GC content of 42,9%. The final coverage was approximately 670 times the average genome size of the *Psychrobacter* genus.

### 2.4 Genome assembly, annotation and analysis

**Fig. 4:**
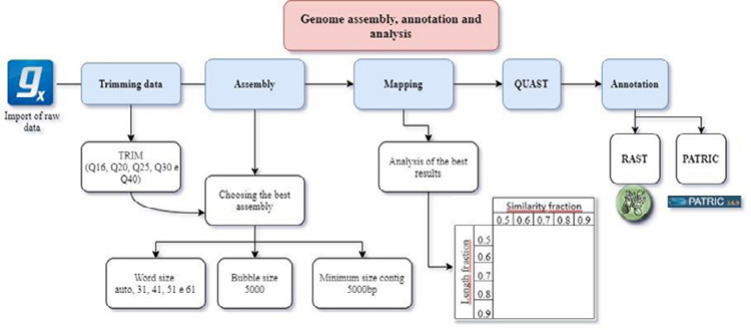
Flowchart of the process performed for cleaning, assembly, verification and annotation of the Psychrobacter nivimaris LAMA 639 genome.

Removal of low-quality raw sequences and *de novo* reassembly based on Bruijin graphs were performed using various trimming parameters in CLC Genomics Workbench software (v 6.5.2) (Fig. 4). The assemblies were analyzed with different parameters and compared by the Quality Assessment Tool for Genome Assemblies (QUAST) [19]. Once the genome was assembled and chosen, the completeness and contamination of the sequence were evaluated using CheckM software [6]. The chosen annotation was performed using Classic Rapid Annotation System Technology (RAST) [20] with disabled filters. Subsequently, it was annotated in the PathoSystems Resource Integration Center (PATRIC) [21] using the RASTtk database [22].

### 2.5 Prospecting of molecules and enzymes of biotechnological interest

Secondary analysis was performed with several different bioinformatics approaches. Searches in the National Center for Biotechnology Information (NCBI) nonredundant (Nr) and Swiss-Prot databases were performed from the Basic Local Alignment Search Tool (BLAST) [14], and searches of secondary metabolite and natural product domains were performed by antibiotics & Secondary Metabolite Analysis Shell (antiSMASH) v 5.0 [16] and Natural Product Domain Seeker (NaPDoS), respectively [18]. Finally, for the analysis of the three-dimensional prediction of cured proteins, Protein Homology/analogY Recognition Engine V 2.0 (Phyre2) [11] was employed.

## Ethics Statement

All ethical requirements were observed in the preparation of the publication. The work was not related to the use of human objects and did not include experiments with animals.

## CRediT authorship contribution statement

**Brendon Egon Kormann Staloch:** Formal analysis, Investigation, Data curation, Writing – review & editing. **Henrique Niero:** Data curation, Visualization, Validation. **Robert Cardoso de Freitas:** Methodology, Software, Supervision. **Patricia Ballone:** . **Fernanda Rodrigues-Costa:** . **Daniela Barretto Barbosa Trivella:** Investigation, Validation. **Andréa Dessen:** Funding acquisition, Investigation. **Marcus Adonai Castro da Silva:** Resources, Methodology. **André Oliveira de Souza Lima:** Conceptualization, Methodology, Funding acquisition, Project administration, Writing – original draft.

## Declaration of Competing Interest

The authors declare that they have no known competing financial interests or personal relationships which have or could be perceived to have influenced the work reported in this article.
